# Praziquantel-Solid Lipid Nanoparticles Produced by Supercritical Carbon Dioxide Extraction: Physicochemical Characterization, Release Profile, and Cytotoxicity

**DOI:** 10.3390/molecules24213881

**Published:** 2019-10-28

**Authors:** Luciana N. Andrade, Daniele M.L. Oliveira, Marco V. Chaud, Thais F.R. Alves, Marcelo Nery, Classius F. da Silva, Joyce K.C. Gonsalves, Rogéria S. Nunes, Cristiane B. Corrêa, Ricardo G. Amaral, Elena Sanchez-Lopez, Eliana B. Souto, Patrícia Severino

**Affiliations:** 1 Laboratory of Nanotechnology and Nanomedicine, Institute of Technology and Research, Aracaju SE 49032-490, Brazil; luciana.nalone@hotmail.com (L.N.A.); marcelonery.pg@gmail.com (M.N.); 2 School of Pharmacy, University Tiradentes, Aracaju SE 49032-490, Brazil; 3 Laboratory of Biomaterials and Nanotechnology, University of Sorocaba—UNISO, Sorocaba SP 18023-000, Brazil; marco.chaud@prof.uniso.br (M.V.C.);; 4 Laboratory of Biotechnology and Natural Products, Federal University of São Paulo, Diadema SP 09913-030, Brazil; classiusferreira@gmail.com; 5 Federal University of Sergipe, São Cristóvão SE 49100-000, Brazilrogeria.ufs@hotmail.com (R.S.N.); crisbani@gmail.com (C.B.C.); ricardoamaral23@hotmail.com (R.G.A.); 6 Department of Pharmacy, Pharmaceutical Technology and Physical Chemistry, and Institute of Nanoscience and Nanotechnology (IN2UB), Faculty of Pharmacy, University of Barcelona, 08028 Barcelona, Spain; esanchezlopez@ub.edu; 7 Faculty of Pharmacy, University of Coimbra (FFUC), Pólo das Ciências da Saúde, Azinhaga de Santa Comba, 3000-548 Coimbra, Portugal; 8 CEB—Centre of Biological Engineering, University of Minho, Campus de Gualtar, 4710-057 Braga, Portugal; 9 Tiradentes Institute, 150 Mt. Vernon St., Dorchester, MA 02125, USA

**Keywords:** praziquantel, solid lipid nanoparticles, supercritical fluid, fibroblasts cell line

## Abstract

Solid lipid nanoparticles (SLNs) can be produced by various methods, but most of them are difficult to scale up. Supercritical fluid (SCF) is an important tool to produce micro/nanoparticles with a narrow size distribution and high encapsulation efficiency. The aim of this work was to produce cetyl palmitate SLNs using SCF to be loaded with praziquantel (PZQ) as an insoluble model drug. The mean particle size (nm), polydispersity index (PdI), zeta potential, and encapsulation efficiency (EE) were determined on the freshly prepared samples, which were also subject of Differential Scanning Calorimetry (DSC), Fourier-Transform Infrared Spectroscopy (FTIR), drug release profile, and in vitro cytotoxicity analyses. PZQ-SLN exhibited a mean size of ~25 nm, PdI ~ 0.5, zeta potential ~−28 mV, and EE 88.37%. The DSC analysis demonstrated that SCF reduced the crystallinity of cetyl palmitate and favored the loading of PZQ into the lipid matrices. No chemical interaction between the PZQ and cetyl palmitate was revealed by FTIR analysis, while the release or PZQ from SLN followed the Weibull model. PZQ-SLN showed low cytotoxicity against fibroblasts cell lines. This study demonstrates that SCF may be a suitable scale-up procedure for the production of SLN, which have shown to be an appropriate carrier for PZQ.

## 1. Introduction

Research in pharmaceutical nanotechnology has focused on the development of modified release nanoparticles for the administration of drugs already used in clinical settings, for which several types of drug delivery systems have been proposed in the literature. These systems might promote the higher concentration of the active substance at the target sites, reducing the toxicity on non-specific tissues.

Due to their high degree of biocompatibility and limited toxicity in vitro and in vivo [1,2,3], solid lipid nanoparticles (SLNs) are being studied for various administration routes to improve drug solubility (and thereby its bioavailability) [2,4] and reduce the risk of adverse side reactions [5,6]. The main production methods of SLNs are based on high homogenization and solvent evaporation/extraction [7,8,9].

Supercritical fluid (SCF), based on the solvent extraction of oil-in-water (o/w) emulsions using supercritical carbon dioxide (CO_2_), has been proposed to produce micro/nanoparticles with a narrow size distribution, better flowability, less residual organic solvent, and with high encapsulation efficiency for different types of drugs (e.g., ketoprofen, indomethacin, camptothecin) [10,11,12]. The final material is reported to be a dried powder that facilitates the production of improved liquid or solid drug formulations, while the technique is described as environmentally friendly and with the potential to be scaled-up [13].

In the present work, we describe the development of a new SLN formulation produced by SCF to load praziquantel (PZQ) for oral administration. The selected model drug is included in the Biopharmaceutical Classification System (BCS) group II, as it shows low solubility and high permeability. Due to its low oral bioavailability and bad taste, the classically available formulations are unsuitable for children [14,15]. The developed PZQ-SLNs were characterized for their mean particle size, polydispersity index, zeta potential, encapsulation efficiency, drug release, and cytotoxicity. The solid crystalline lipid matrix loading PLZ, and absence of chemical interactions between the drug and the lipid, have been confirmed by Differential Scanning Calorimetry (DSC) and Fourier-Transform Infrared Spectroscopy (FTIR), respectively.

## 2. Results and Discussion

Many drugs currently in use in clinics exhibit high lipophilicity, low bioavailability, high toxicity, and high sensitivity under some environmental conditions. Given these limitations, drugs need to be processed using innovative techniques such as nanoencapsulation. Conventional encapsulation techniques use a substantial volume of organic solvents and operate at extremely high or low temperatures, resulting in formulations with high polydispersity and high scale-up limitations. Due to these disadvantages, an alternative approach was proposed to produce nanoparticles by SCF, which is environmentally friendly, easier to scale up, and generates low organic residues. In the production of our SLNs, DCM has been used as organic solvent. The advantage of SCF is however the possibility of easy recovery of the supercritical solvent after the extraction process, just by adjusting the pressure and temperature, being continuously recycled. This approach eliminates one of the most expensive steps of conventional extraction processes, which is the separation between extracted products and the organic solvent. Also, the handling of large amounts of polluting organic solvents poses additional drawbacks with respect to environmental control, whether of air quality, liquid effluent, or solid waste. A positive outcome of the efficient separation between solute and supercritical solvent is the high purity product that is obtained, as the process leaves no solvent residues in the final product. Polymeric nanoparticles, metal nanoparticles, nanostructured microparticles, nanoporous materials, and SLNs have been produced using SCF [16].

SLNs are superior drug delivery systems attributed to their biocompatibility, modified-release properties, and high loading capacity for poorly water-soluble drugs. In this work, PZQ was used as a model drug in SLNs produced by SCF. Blank SLNs (without PZQ) and PZQ-SLNs showed mean particle sizes of 24.60 ± 2.45 nm and 23.78 ± 4.52 nm, respectively, without any statistical difference (*p* < 0.05). PdI was also considered in this study as one of the parameters to evaluate the quality of the SLN production by SCF. PdI measures the deviation of the autocorrelation function from that of dispersions having monodisperse nanoparticles; low PdI values translate to high-quality dispersions. Blank SLNs and PZQ-SLNs showed a unimodal distribution, with PdI values of 0.50 ± 0.22 and 0.55 ± 0.12, respectively, which are appropriate for oral administration (i.e., around 0.4 to 0.5). According to Danaei et al., a PdI of 0.3 or below in lipid nanoparticle populations is considered to be acceptable when intended for parenteral administration [17]. As oral administration does not have the same size restrictions, a higher PdI is tolerated by this pathway. Although the last edition of the FDA’s “Guidance for Industry” concerning liposome drug products emphasizes the importance of size and size distribution as “critical quality attributes (CQAs),” as well as essential components of stability studies of these products, the document does not mention the criteria for an acceptable PdI. More specific standards and guidelines for the acceptability of product PdI vary among the different applications (e.g., food, cosmetic, pharmaceutical, etc.) and different administration routes.

Zeta potential is a measure of the electrical voltage difference between the surface colloidal suspension. It gives information about the stability of a colloidal dispersion; the recommended value is higher than +/−30 mV. The ZP values of blank SLN and PZQ-SLN values were of −29.52 ± 0.52 and −27.25 ± 0.59, respectively. The negative ZP values are attributed to the negative soybean lecithin that acts as surfactant stabilizing SLNs.

The EE% of PZQ-SLN reached 88.37%, due to the lipophilicity of PZQ. The maximum loading capacity obtained for PZQ in SLN was 17.64 mg.

Xie et al. encapsulated PZQ in SLNs produced by hot homogenization and ultrasonication method, reaching an EE% of 62.17±6.53%. Jelowdar et al. produced PZQ-SLNs with EE of 59% and confirmed their efficiency in the reduction of *echinococcosis* cysts in vivo [18].

**Figure 1:**
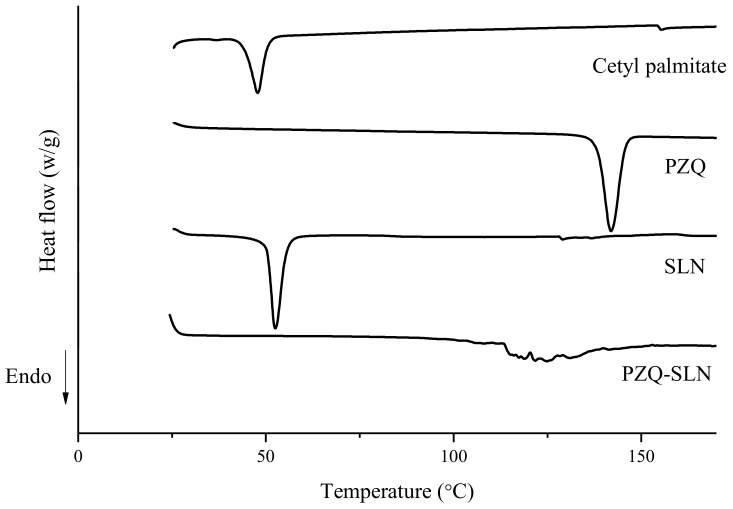
Differential Scanning Calorimetry (DSC) curves of praziquantel solid lipid nanoparticle (PZQ-SLN), SLN, PZQ, and cetyl palmitate.

DSC technique has been widely used to obtain information about the purity of the standard reference; thermal transitions reflect the physical and chemical processes that are associated with a release or absorption of heat. Such transitions include melting, glass transition, polymorph conversion, crystallization, desolvation, dehydration, and chemical reactions [19]. Furthermore, DSC measures the changes that take place in a material with a change in temperature. Such changes are monitored by measuring the heat flow associated with the heating of a sample comparing it to the reference while they are subjected to the same controlled temperature program. The DSC thermograms of PZQ-SLN, SLN, PZQ, and cetyl palmitate are shown in Figure 1.

The DSC curve of the free PZQ exhibited an endothermic peak of relative intensity at 141.96 °C. This value is close to that recorded by Chaud et al. [20] as being the melting temperature of the drug. A single endothermic event (ΔH = −158.89 J/g), at 47.84 °C, was recorded for the bulk cetyl palmitate. The melting of this lipid may occur at different temperatures depending on the type of polymorphs present. Low temperatures (e.g., close to 40 °C) are attributed to the presence of α-form, followed by the metastable β’-form, and then by the most stable β-form at temperatures close to 50 °C. Waxes as cetyl palmitate are commonly recrystallized in the metastable β’-form. During the cooling of the melted lipid, the recrystallization of the lipid matrix occurs for the solidification of the core and formation of SLNs. These changes result in phase transitions of the structural order, such as crystalline state and enthalpy, due to the formation of a new structure promoted by the method of production. This new structure may also be the reason for values of PdI obtained for the SLN dispersions. When analyzing the DSC curves of SLNs, it was shown that the production procedure provided a slightly higher temperature shift (Tpeak = 52.64 °C) with enthalpy reduction (ΔH = −80.04 J/g) when compared to bulk cetyl palmitate, which translates the formation of solid, yet less crystalline, SLN matrices.

In PZQ-SLN thermograms, a new DSC curve profile has been recorded, exhibiting a set of higher intensity peaks at 124.95 °C, and an even more significant reduction for enthalpy (ΔH = −38.80 J/g). The thermal behavior of the loaded SLNs, mainly in the enthalpy values, suggests that a large polymorphic transformation is influencing these changes, attributed to the use of supercritical fluid as the production methodology, providing significant structural changes and causing a decrease in the degree of SLN crystallinity. The decrease of enthalpy of the PZQ-SLNs and absence of the PZQ suggest, respectively, that the drug is loaded in the particles and that it is solubilize in the selected solid lipid. The low degree of crystallinity contributes to increase the loading of the lipid matrices with PZQ, and thereby high EE%. The complete solubilization of the drug in the melted cetyl palmitate of PZQ-SLN was confirmed by suppression of the PZQ peak at 141.96 °C in the thermogram of the drug-loaded particles.

**Figure 2:**
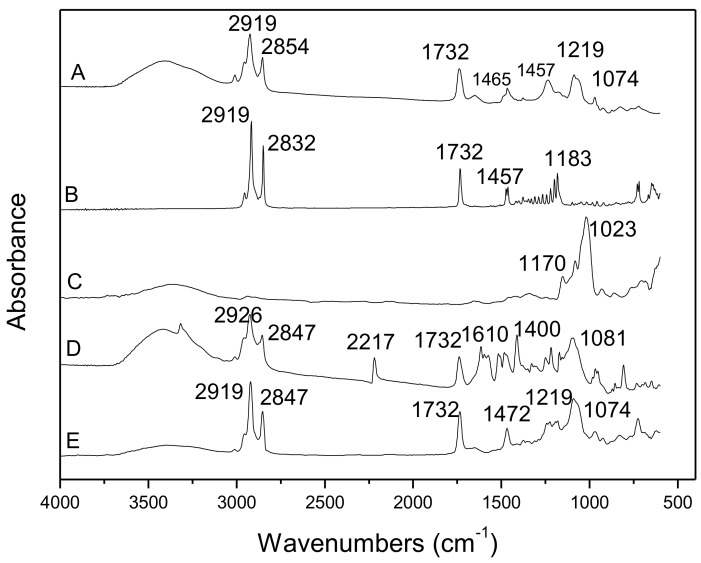
Fourier-Transform Infrared Spectroscopy (FTIR) spectra of (**A**) soybean lecithin; (**B**) cetyl palmitate; (**C**) PZQ; (**D**) PZQ- SLN; and (**E**) SLN.

FTIR allows for the identification of functional groups present in the materials. The observation of the vibration spectrum of the encapsulated drug allows evaluating the type of interaction that occurs between the drug and the matrix material, since the vibrations of the atoms involved in this interaction can suffer changes of frequency and intensity. FTIR spectra of soybean lecithin, cetyl palmitate, PZQ, PZQ-SLN, and SLN are shown in Figure 2.

For PZQ, the wide band in the region 3600–3400 cm^−1^ indicates a characteristic stretching of the O–H group and can be attributed to the presence of water molecules. It shows major peaks from the groups of molecules such as carbonyl stretch vibrations –C=O (1630 cm^−1^), –CH, –CH2, –CH3 (2900–3000 cm^−1^), and C–N stretch vibrations (1000–1350 cm^−1^). Similar results have been reported by Chaud et al. [21]. Other characteristic bands occur between 1244 and 1066 cm^−1^ attributed to the axial deformations C–N, symmetrical angular plane C–H cyclic structure at 1411 cm^−1^. The FTIR results for PZQ are in agreement with those described in the literature [22,23]. Soybean lecithin (**A**) showed peaks for –CH, –CH_2_, –CH_3_ elongations (2850–2850 cm^−1^), carbonyl stretch vibrations –C=O (1700–1734 cm^−1^), stretching of C–O (1025–1244 cm^−1^), and OH– stretching vibrations (3100–3418 cm^−1^), which were broad. The peak at 2217 is associated with the formation of a carbon–nitrogen bond between PZQ and soy lecithin [24], which appears only in D (Figure 2). The results demonstrate that there was no chemical interaction between the drug and the soybean lecithin because their spectra shape and position were not significantly different. The formulation of SLN (**E**) and SLN loaded PZQ (**D**) showed peaks resulting from the simple superposition of their separated components in the FTIR. The spectral analysis indicated that PZQ-SLN (**D**) specific functional groups on the surface of the nanoparticles exhibit almost the same chemical characteristics as cetyl palmitate (**B**) and the trapped drug exhibits its main characteristic peaks. The present study suggests that there were no molecular interactions that could modify the chemical structure of PZQ.

**Figure 3:**
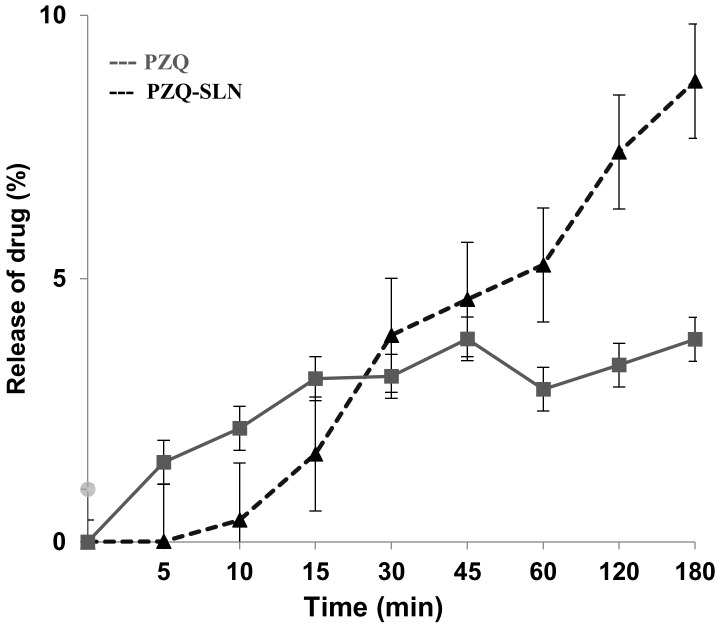
In vitro drug release profile of free PZQ and PZQ-SLN.

The in vitro behavior of the isolated PZQ drug release and PZQ-SLN was investigated using a dialysis membrane in phosphate buffer solution at pH 7.0 (37 ± 0.2 °C). Figure 3 shows the obtained release profiles.

PZQ exhibited a maximum release rate of 3.85% drug in 8 h, while the release profiles of PZQ-SLN indicated a release of 7.64%. There was a statistical difference between the evaluated samples, demonstrating that a release of the PZQ from SLN occurred in a more controlled pathway when compared to the isolated PZQ. The release modelling was analyzed by finding the R^2^ value for each kinetic model, namely, Baker and Lonsdale, Peppas, Hixon and Crowell, Higuchi, Square Root, First Order, and Weibull. The parameters corresponding to the release data obtained. The parameters model of Korsmeyer-Peppas and Weibull 5 were the best (R^2^ = 1.0), as shown in Table 1.

The Weibull model can be applied successfully on almost all types of release curve and exponentially relates the fraction of the released drug. The Weibull equation expresses the cumulative amount of drug as a function of time and should be applied to the data of the first 63.2% drug release. On the other hand, the Korsmeyer-Peppas model takes into account that the mechanism of drug release does not follow Fick’s law and follows an anomalous behavior described by Equation (1).

(1) $$\frac{M_{t}}{M_{\infty }}=\;kt^{n}$$

where M_t_ is the amount of drug released in a given time t, M_∞_ is the amount of drug released at an infinite time, k is the release kinetic constant, and *n* is the release exponent. The value of *n* is related to the geometric form of the release system and determines the release mechanism. The Kornmeyer-Peppas model is generally used to analyze the release of a drug from a matrix when it is not well known or when one or more types of phenomena are involved.

PZQ-loaded SLNs presented release kinetics following two mathematical models, being indicative of the structural heterogeneity of the formulation, which possibly demonstrates the SLN formation coated by multiple layers of surfactant, also demonstrated by the PdI obtained for the dispersion. The lipid used for the preparation of SLNs plays a key role in the design of release kinetics, as the lipid is responsible for the encapsulation of the drug in the matrix of the particles. Our results suggest that cetyl palmitate used in the formulation forms a polymorphs structures which control the drug outlet of the SLNs. However, as the drug dissolves, the porosity of the matrix increases, so that the drug can be released. In addition, the use of Tween^®^20 as surfactant, causes the compacted systems to be dissolved by progressive erosion, releasing PZQ. Tween^®^20 is able to promote drug release because of increased contact of the drug in water, promoting a greater dispersion of the drugs, besides favoring the release of the same by disintegration.

**Figure 4:**
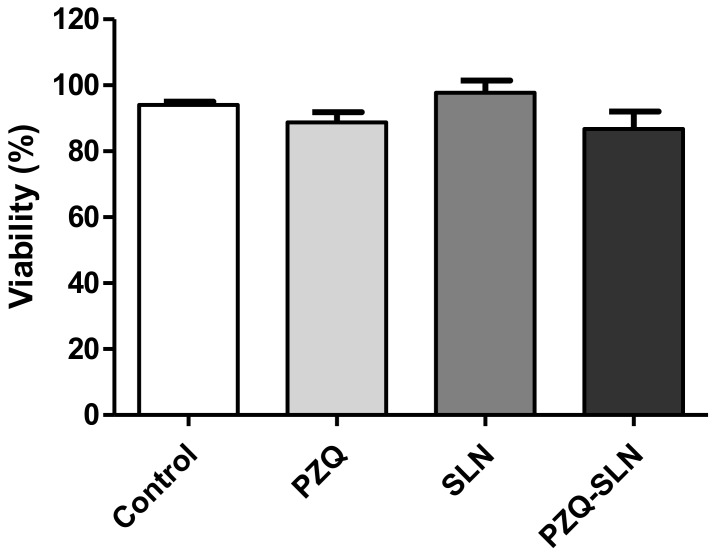
Effect of praziquantel, SLNs, and PZQ-SLNs supercritical in the evaluation of the viability of human L929 fibroblasts determined by the MTT assay after 24 h of incubation. The negative control (C) was treated with the vehicle used to dilute the drug (DMSO 5%). The data correspond to the mean ± SEM of four independent experiments.

In vitro cytotoxicity was performed using human fibroblast L2929 as a cellular model. As reported in the scientific literature, although the lipids and surfactants used for the production of SLNs are of GRAS status, the cytotoxicity of these raw materials is dependent on the concentration and type of cell model. The administration route is also decisive to set the limits of acceptance. Besides the raw materials, the cytotoxicity of SLNs is dependent on the size and surface properties, as well as on the concentration of the particles in contact with the cells. The cytotoxicity profile of both blank and PZQ-SLNs should be obtained. The data are shown as a percent of cellular viability (Figure 4). The cell viability results demonstrate that free PZQ did not kill normal human fibroblasts. Indeed, cellular viability was greater than 88%. Moreover, blank SLNs (97.78%) and PZQ-SLNs (86.77%) also did not show any toxicological activity. The results indicate the safety and biocompatibility of both SLNs and PZQ-SLNs with fibroblasts cells. Any cytotoxic effect of particles can be due to their adherence to the cell membrane, particle internalization and/or degradation of products in the cell culture medium or inside the cells. However, the susceptibility of different cell types can be different for different particulate carriers. Because the SLNs contain natural lipids, they should be well-tolerated by living organisms [25].

## 3. Materials and Methods

### 3.1. Materials

PZQ was provided by Henrifarma^®^ (Cambuci, São Paulo, Brazil). Soybean lecithin (Lipoid^®^ S75) was purchased from Lipoid GmbH (Ludwigshafen, Germany). Cetyl palmitate was donated as a gift from Croda (Campinas, Brazil). All other reagents were bought from Sigma-Aldrich (Sintra, Portugal). Double-distilled water was used after filtration in a Millipore^®^ system (home supplied).

### 3.2. Production of SLN

The oil phase, composed of solid lipid (cetyl palmitate, 150 mg), drug (PZQ, 20 mg), surfactant (Tween 20, 100 mg), and co-surfactant (soy lecithin, 150 mg), was solubilized in dichloromethane (50 mL) using magnetic stirring (Thermo Scientific Cimarec Stirring Hot Plate, 7 × 7 Ceramic; 120 VAC) for 30 min. SLN were then placed in a continuous extraction method from Waters Corporation (SAS-RESS Combined System, Pittsburgh, PA, USA). The method consisted on the introduction of the organic solution into an extraction column from the top at a constant flow rate of supercritical CO_2_ (at a constant pressure of ~80 bar and 35 °C) as a countercurrent. The residence time required for SLN production was 2 min. The solvent-borne CO_2_ was carried out in the extraction column for solvent recycling, and SLNs were then collected in the vessel was collected for further characterization. The equipment allows the production of size batches from 100 mL to 5000 mL. The batch size of our PZQ-SLNs was 100 mL.

### 3.3. Mean Particle Size, Polydispersity Index, and Zeta Potential

The mean particle size (z-Ave), polydispersity index (PdI), and zeta potential (ZP) of the SLNs were determined by photon correlation spectroscopy (Malvern Zetasizer, Nano Z-S; Malvern Instruments, Worcestershire, UK). Measurements were performed at a 90-degree angle at 25 °C.

### 3.4. Encapsulation Efficiency (EE) and Loading Capacity (LC)

The encapsulation efficiency (EE) and the loading capacity (LC) of the PZQ into SLNs were determined indirectly, by measuring the concentration of free, unloaded PZQ in the aqueous medium of the nanoparticles. Briefly, approximately 1 g of PZQ-SLNs was weighed and ultra-centrifuged for 40 min at 4 °C at 16,000 rpm (Biofuge Strato, Hong Kong). The amount of non-loaded PZQ in the supernatant was determined using ultraviolet (UV) spectrophotometry with the detection wavelength set at 264 nm [15]. EE (%) and LC (%) were calculated in triplicate using Equations (2) and (3) [2,4].

(2) $$\mathrm{EE}\;\left(\%\right)\;=\;\frac{\mathrm{Total}\;\mathrm{amount}\;\mathrm{of}\;\mathrm{PZQ}-\mathrm{PZQ}\;\mathrm{in}\;\mathrm{supernatant}}{\mathrm{Total}\;\mathrm{amount}\;\mathrm{of}\;\mathrm{PZQ}}\times 100$$

(3) $$\mathrm{LC}\;\left(\%\right)=\;\frac{\;\mathrm{Total}\;\mathrm{amount}\;\mathrm{of}\;\mathrm{PZQ}-\mathrm{PZQ}\;\mathrm{in}\;\mathrm{supernatant}}{\mathrm{Total}\;\mathrm{amount}\;\mathrm{of}\;\mathrm{PZQ}}\;\times 100$$

### 3.5. Differential Scanning Calorimetry (DSC)

The analysis of the solid state of the lipid matrices (SLNs) was performed using a DSC TA 2920 (Newcastle, DE, USA). The samples were placed in the hermetic aluminum pans, and the experiments were performed under a nitrogen gas flow at a heating rate of 10 °C/min over a temperature range of 25–350 °C [7].

### 3.6. Fourier-Transform Infrared Spectroscopy (FTIR)

The Fourier-transform infrared spectroscopy (FTIR) spectra of the SLNs, PZQ, and PZQ-SLNs were recorded using potassium bromide discs on a Nicolet 5 SXC FTIR Spectrophotometer (Madison, WI, USA). The potassium bromide discs were prepared by compressing the corresponding powder.

### 3.7. In Vitro Drug Release Studies

The in vitro drug release assay was determined by the dialysis bag method with some adaptations [15]. The equivalent of 2 mL of the PZQ-SLN formulation (described in 3.2) and 2 mL of PZQ solution (0.8 mg PZQ dissolved in 2 mL water) were placed in independent dialysis bags (L 50 mm x W 25 mm x D 16 mm, InLab, São Paulo, Brazil). Both dialysis bags were submerged in 10 mL water and 40 mL phosphate buffer solution at 37 ± 0.2 °C, pH 7.0 ± 0.3 and under constant magnetic stirring (200 rpm). Then, at pre-determined time-intervals (5–180 min), a volume of 200 μL of the release medium was collected, filtered through a membrane of 0.45 μm pore size, and analyzed in a UV spectrophotometer (UV-Vis Spectrophotometer, Model UV2600 Shimadzu Corporation, Tokyo, Japan). The same volume of fresh phosphate buffer solution at the same temperature was added immediately to keep the release volume constant and guarantee sink conditions. The method was validated using the spectrophotometry technique in the ultraviolet (UV) region according to Resolution 899 in 2003 by the National Agency of Sanitary Surveillance (ANVISA). The drug release studies were performed in triplicate. Finally, the best model for evaluating the rate of drug release was determined using the Sigmaplot software (Systat Software Inc., San Jose, CA, USA).

### 3.8. Cell Viability Assay in L929 Cells

The human fibroblast cell line L929 was used in testing the free PZQ, SLNs, and PZQ-SLNs at a concentration of 30 μg/mL each, based on the ISO 10993-5 [26]. L929 cells were seeded in 96-well culture plates (2 × 10^4^ cells/well) and cultured in DMEM media containing NaHCO_3_, ampicillin, streptomycin, and supplemented with 10% fetal bovine serum. After 24 h of treatment, the wells were washed, and the cells were exposed to 10 μL of the 5 mg/mL 3-(4,5-dimethylthiazol-2-thiazolyl)-2,5-diphenyltetrazolium bromide (MTT) solution, which was left to react for 3 h [27]. The MTT solution was then removed, and 80 µL of dimethylsulfoxide (DMSO) was added to each well. The plates were shaken gently for 10 min, and then the absorbance was measured in an ELISA microplate reader at 570 nm.

### 3.9. Statistical Analyses

The data obtained were expressed as the mean ± standard error of the mean (SEM), and the differences between the experimental groups were evaluated using one-way analysis of variance (ANOVA) followed by the Tukey’s post hoc test. Significant values with *p* < 0.05 were considered. All statistical analyses were performed using the GraphPad Program (Intuitive Software for Science, San Diego, CA, USA).

## 4. Conclusions

The production of PZQ-SLNs by SCF shows promising results when using cetyl palmitate as solid lipid. The method produced small-sized SLN, of polydispersity appropriate for oral administration, with a modified release profile and non-toxic against fibroblasts. A high encapsulation efficiency was reached for praziquantel in cetyl palmitate SLNs.

## Figures and Tables

**Table 1:** Coefficient of in vitro release of different mathematical models for formulations PZQ, SLN, and PZQ-SLN.

	Square r Value (r^2^)	
	*PZQ-SLN*	*PZQ*
Mathematical Models		
Baker and Lonsdale	0.8851	−0.4559
Korsmeyer-Peppas	1.0	0.8215
Hixon and Crowell	0.9999	−2.2413
Higuchi, Square Root	0.8881	−0.4665
First Order	0.9999	−2.2292
Weibull	1.0	1.0
